# Remote spatial memory in aging: all is not lost

**DOI:** 10.3389/fnagi.2012.00025

**Published:** 2012-09-13

**Authors:** R. Shayna Rosenbaum, Gordon Winocur, Malcolm A. Binns, Morris Moscovitch

**Affiliations:** ^1^Neuroscience Graduate Diploma Program, Department of Psychology, York UniversityToronto, ON, Canada; ^2^Rotman Research Institute, BaycrestToronto, ON, Canada; ^3^Department of Psychology, University of TorontoToronto, ON, Canada; ^4^Department of Psychiatry, University of TorontoToronto, ON, Canada; ^5^Department of Psychology, Trent UniversityPeterborough, ON, Canada; ^6^Dalla Lana School of Public Health, University of TorontoToronto, ON, Canada; ^7^Department of Psychology, BaycrestToronto, ON, Canada

**Keywords:** aging, hippocampus, landmark recognition, mental navigation, recollection, remote memory, route learning, spatial memory

## Abstract

The ability to acquire and retain spatial memories in order to navigate in new environments is known to decline with age, but little is known about the effect of aging on representations of environments learned long ago, in the remote past. To investigate the status of remote spatial memory in old age, we tested healthy young and older adults on a variety of mental navigation tests based on a large-scale city environment that was very familiar to participants but rarely visited by the older adults in recent years. We show that whereas performance on a route learning test of new spatial learning was significantly worse in older than younger adults, performance was comparable or better in the older adults on mental navigation tests based on a well-known environment learned long ago. An exception was in the older adults' ability to vividly re-experience the well-known environment, and recognize and represent the visual details contained within it. The results are seen as analogous to the pattern of better semantic than episodic memory that has been found to accompany healthy aging.

## Introduction

Healthy aging is characterized by a variety of neural changes, with the hippocampus among the most prominent brain structures to be affected (e.g., Jernigan et al., 2001; Raz et al., 2005; Park and Reuter-Lorenz, 2009). These changes are accompanied by difficulties in forming and retaining new spatial memories of allocentric relations among locations (e.g., Barnes, 1979; Winocur and Gagnon, 1998; Head and Isom, 2010; Harris and Wolbers, 2012). Whereas the hippocampus is needed to support such spatial representations (e.g., O'Keefe and Dostrovsky, 1971; Morris et al., 1982; Wolbers and Büchel, 2005; Nedelska et al., 2012; but see Corkin, 2002), it does not appear to be needed for all aspects of remote spatial memory for environments encountered long ago^1^ (Rosenbaum et al., 2000, 2004, 2007; Maguire et al., 2006). However, there is little information about the effects of aging on remote spatial memory. The current study examines the integrity of remote spatial memory in healthy young and older adults.

The ability to flexibly represent the external world in order to navigate efficiently between spatial locations in both new and familiar environments is essential for independent living. Despite its importance, there is a long-standing debate about the neural substrate of allocentric spatial memory for large-scale environments, particularly those that were experienced long ago. Central to this debate is the role of the hippocampus. A long-standing theory is that the hippocampus is always necessary for supporting allocentric spatial memory (“cognitive maps”) to navigate an environment, no matter how long ago that memory was acquired, as opposed to egocentric representations of the environment within body-centered coordinates (O'Keefe and Nadel, 1978; Bird and Burgess, 2008). A derivative of this theory views scene construction as the central role of the hippocampus. Scene construction involves the retrieval and integration of relevant details into a coherent spatial framework within which details of personal memories can be re-experienced and manipulated into imagined new experiences (Byrne et al., 2007; Hassabis et al., 2007; Hassabis and Maguire, 2009). Based on these theories, one might predict that both recent and remote spatial memory and navigation that rely on allocentric cues would be affected by hippocampal damage. An alternative prediction is that deficits in scene construction may only impair the detailed perceptual representations of an environment, such as is required to recall or imagine scenes, but not for navigation.

Another view that may be considered complementary is that the hippocampus has a time-limited role in representing coarse, schematic, or semantic-like aspects of spatial memory, but is needed always for representing rich, detailed episodic-like aspects (Rosenbaum et al., 2001; Moscovitch et al., 2005; Winocur et al., 2010a). By schematic representations, we mean map-like representations that contain information about landmarks, their location, and relation to one another, which are needed for navigation^2^. From our view, this schematic representation is impoverished with respect to perceptual details that are incidental to navigation, such as the appearance of houses (Rosenbaum et al., 2000) and even of the landmarks themselves. We consider these more detailed aspects as part of the representation that allows for a rich re-experiencing of an environment. The distinction between schematic and detailed spatial representations, which may or may not be orthogonal to allocentric vs. egocentric frameworks, is based on findings from lesion and neuroimaging studies. Amnesic people with hippocampal lesions show evidence of relatively preserved navigation but impaired memory for perceptual details of environments learned long ago (Teng and Squire, 1999; Rosenbaum et al., 2000, 2005; Maguire et al., 2006). These findings are consistent with evidence from neuroimaging studies showing a relative lack of hippocampal activation for mental navigation in the same environments (Rosenbaum et al., 2004, 2007; Hirshhorn et al., 2012). The hippocampus is known to suffer structural and functional decline with age. Similar to hippocampal amnesic patients, older adults show a decline in autobiographical episodic memory for both recent and remote events and associated recollection processes, but relative preservation of semantic memory and associated familiarity (e.g., Davidson and Glisky, 2002; Levine et al., 2002; Piolino et al., 2006).

The changes in declarative/relational memory that do occur appear to relate to significant atrophic changes in hippocampal volume associated with aging (Yonelinas et al., 2007). Aging has also been associated with spatial disorientation in recently encountered environments in both animals and humans in relation to hippocampal volume loss (e.g., Driscoll et al., 2006; Moffat, 2009). A study of older adults suggests that this impairment extends to the re-experiencing of familiar routes from an environment that was traveled extensively in the past, which is correlated with neuropsychological tests of hippocampal function and autobiographical episodic memory (Hirshhorn et al., 2011). Nevertheless, the ability to represent the spatial distance between landmarks located in the same environment does not appear to be affected, though comparison of the older adults' remote spatial memory performance was not made with a younger group. In a more direct investigation, aged rats with prior exposure to a complex “village” environment showed significantly better memory for allocentric spatial relations among locations contained within that environment than age-matched rats who were naïve to the environment (Winocur et al., 2010a). The experienced aged rats performed slightly worse than they had as young rats during initial training, but better than a separate group of young rats not previously exposed to the environment, indicating both preserved remote spatial memory and impaired new spatial learning in old age.

Overall, the findings suggest that spatial representations of well-learned environments formed long ago are relatively impervious to the effects of aging and, together with earlier findings of similar preservation in young rats with hippocampal lesions (Winocur et al., 2005, 2010a), do not depend on the hippocampus. To our knowledge, however, systematic examination of remote spatial memory in young vs. older adults has not been attempted in humans. If the hippocampus, which functionally declines with age, is not needed for various aspects of remote spatial memory, particularly those which support navigation, then aging should not affect performance on spatial memory tasks based on an environment that was extensively navigated in the past, even if it affects spatial learning in newly encountered environments. Retention and retrieval of perceptual details of an environment, whether experienced recently or long ago, however, should be impaired in older adults as these always are dependent on the hippocampus. The data presented in the current study support these predictions.

## Materials and methods

### Participants

A group of 14 healthy older adults aged 65–85 years (half male; 13 right-handed) were recruited from the Baycrest participant pool for monetary compensation. Comparisons were made with a group of 14 young adults aged 18–30 years (half male; 13 right-handed) recruited from the Baycrest participant pool for monetary compensation and the York University Undergraduate Research Participant Pool for course credit. Demographic information and other descriptive data are summarized in Table 1. Participants were matched for years of education, fluent in English, free from a history of neurological and psychiatric illness, and lived in Toronto for a minimum of 10 years. None of the older participants met criteria for dementia based on the MMSE or MoCA. The study received approval from the York University and Baycrest research ethics boards.

**Table 1:** **Demographic characteristics of the younger and older participants**.

		**Younger**		**Older**	
		**Mean**	**SD**	**Mean**	**SD**
Age (years)		22.21	4.00	72.21	6.31
Education (years)		14.36	1.15	15.29	1.98
Living in Toronto (years)		18.21	4.66	50.5	16.16
Visit frequency^*a*^		3.82	1.38	2.71	1.12
Navigation ability^*b*^	New environments	3.54	1.15	4.04	0.89
	Familiar environments	4.43	0.85	4.5	0.65

Note: SD, standard deviation.

a Based on scale ranging from 1 to 5, where 1 = no more than once a year, 2 = 1 to 2 times per year, 3 = once a month, 4 = once a week, and 5 = more than once a week.

b Based on subjective ratings on a Likert scale, ranging from 1 (difficulty navigating/always disoriented) to 5 (navigates with ease/never disoriented).

### Materials and procedure

Participants' remote spatial memory was tested for mental navigation amid landmarks and for the visual identity of those landmarks located in a city environment (downtown Toronto, approximately 5 square km). The environment was experienced approximately 2–3 times per week for at least 10 years by all participants and up to 45 years ago by the older participants. Most of the older participants rarely experienced the environment in the last 5 years. The tasks were designed to simulate the demands of negotiating through large-scale space, with mental navigation tasks varying in terms of their demands on allocentric (tasks 1–4) vs. egocentric processing (tasks 5 and 6; see Ciaramelli et al., 2010, for a detailed rationale for task classification). Comparisons in remote spatial memory performance were made with spatial memory acquisition on a route learning test. Tasks were presented in a fixed order, as follows.

#### Mental navigation tests (Toronto Public Places Test; TPPT)

***Proximity judgments***. In a test of relative distance judgments, participants indicated which of two Toronto landmarks was closest to a third reference landmark. The actual distance among the 10 sets of landmarks for each environment varied from trial to trial, and half of the trials were more demanding (i.e., the difference in distance between the reference landmark and either of the choice landmarks was less than 1 km).

***Distance judgments***. Participants were asked to provide numerical judgments of absolute distance between each of 10 pairs of landmarks located in downtown Toronto in their preferred unit of measure (i.e., km or miles). A sample trial was administered prior to testing in order to give the participants an indication of scale. The actual distances between landmarks were varied and randomly intermixed across trials. The mean deviation of the judged distances from the actual distances in km was calculated for each trial and averaged to derive absolute error scores.

***Vector mapping***. In a test of allocentric distance and head-direction between landmarks, participants were asked to draw arrows indicating the correct distance and direction from a location specified by a mark to an unmarked landmark on 10 maps of downtown Toronto that included lines indicating northern and southern downtown city limits. Deviation of estimates from actual directions in degrees and distances in km was calculated for each trial and averaged to derive absolute error scores.

***Landmark sequencing***. Ten randomly ordered names of landmarks located along a north-south route were presented, and participants were to order the landmarks in the sequence that would be passed during a mental walk of the route.

***Blocked routes***. Participants were asked to simulate taking shortcuts in a task requiring a change of route from the most direct route between a pair of landmarks. There was a total of 5 such trials, each consisting of 2 to 4 choice points at which to turn right or left, for a maximum score ranging from 11 to 16 per participant. Partial points were given for impoverished descriptions of routes that otherwise led to the specified destination. At the end of the task, participants were additionally asked whether they felt that they were remembering the simulated navigation episodes from a first-person perspective (i.e., as being actually involved in the episode; e.g., driving or walking on the streets) or from a third-person perspective (i.e., as being an observer of the episode, or adopting a survey perspective), and whether they had experienced the routes as vivid and rich in detail while mentally navigating them.

#### Landmark appearance

Participants were asked to distinguish between photographs of downtown Toronto landmarks and of buildings that are structurally similar to those located in downtown Toronto but that have never been encountered by the participants. The stimulus set included a total of 25 landmarks and 25 distractors. All photographs were taken from an unobstructed view and were digitally scanned and adjusted for luminance and contrast. For each photograph, participants were to decide if the landmark is familiar, and if so, to identify it by name and location or by some other means if necessary (e.g., type of building, decade in which it was established, function).

#### Baycrest route learning test

A route learning test previously found to be sensitive to hippocampal function in Alzheimer's disease (Rosenbaum et al., 2005) was adapted for the present study to assess spatial acquisition. Participants were taken on a novel route through two floors of Baycrest, where the Rotman Research Institute is located. Four of the older participants had visited Baycrest prior to the current study, and none had traversed the particular test route. There were 16 choice points along the route where participants had to decide whether to turn right or left or to continue straight. The route covered three floors of the hospital, and participants relied on an elevator to travel between floors. Four legs of the route included large windows with views to distal outdoor cues; the remainder of the route did not include views to the outside.

***Procedure***. The learning phase involved a 15-min experimenter-led tour of the route. Participants were instructed to pay attention to the route and its visual features as reminders of where to turn because they would later be asked to lead the experimenter on the same route. While on the tour, the experimenter did not point out landmarks or turns, other than the destination floor when the elevator was taken. During the test phase, the participant led the experimenter through the route, with errors recorded and corrected to maintain the flow of the route. After a 30 min filled break, during which the landmark appearance test was administered, participants were asked to lead the experimenter through the route a final time.

### Statistical analyses

Most of the tasks (Proximity Judgments, Landmark Sequencing, Blocked Routes, Landmark Identification, and Baycrest Route Learning) generated error count data as the outcome variable of interest. For these measures we entered number of errors as the dependent variable in a Poisson regression to examine differences between the younger and older participants. We adjusted the null hypothesis tests using the deviance scaling option to compensate for under- or over-dispersion. An offset variable was included for the Blocked Routes test to accommodate differences in the total number of streets used for each trial per participant. The remaining tasks (Distance Judgments, Vector Mapping, and Landmark Recognition) generated outcome variables with reasonably bell-shaped distributions, and these were entered into an analysis of variance (ANOVA) with Group (young vs. old) as a between-subject factor. In order to examine the effects of differences in exposure to downtown Toronto on the main effect of age, the number of years living in Toronto and frequency of recent visits to the downtown core (i.e., within the past 5 years) were included individually as covariates in all models. Finally, correlations in performance on the experimental measures, stratified by age, were calculated. All hypothesis tests are performed at an alpha level of 5%.

## Results

As indicated by the descriptive data in Table 1, the young adults visited downtown Toronto more frequently within the past 5 years compared to the older adults, whereas the older adults lived in Toronto for a significantly longer time than the young adults. Although each of the variables were included separately as covariates in the analyses, the lack of overlap in groups on the latter variable may present a challenge to interpreting conditional age effects in test performance. Surprisingly, subjective ratings of navigation ability were lower for the young adults compared to the older adults for new environments and indistinguishable for familiar environments. Participants' performance on all spatial memory tests is presented in Table 2.

**Table 2:** **Performance of young and older participants on experimental tasks**.

**Experimental task**		**Young**		**Old**		***p*-value**	***d***	**pd**	**e^β^**
		**Mean**	**SD**	**Mean**	**SD**				
**MENTAL NAVIGATION**									
Proximity	error	1.21	1.42	1.14	0.95	0.88	0.06	−0.06	1.06
Distance	deviation (km)	1.99	2.66	1.04	0.7	0.2	0.49	−	−
Vector	deviation (km)	0.36	0.19	0.28	0.11	0.06^†^	0.51^‡^	−	−
	deviation (°)	17.47	7.29	12.38	5.12	0.007^†^	0.81	−	−
Sequencing	error	1.43	1.45	0.93	1	0.33	0.4	−0.35	1.54
Blocked Route	error	3.64	2.6	2.61	1.06	0.0005^†^	0.52^‡^	−0.28	1.99
**LANDMARK APPEARANCE**									
Recognition	hits − fa	17.64	2.62	13.21	4.56	0.004	1.19	−	−
	hits + fa	26.93	4.5	24.07	5.94	0.04^*^	0.54	−	−
Identification	error	16.25	3.79	13.69	2.68	0.07	0.78	−0.16	0.77
**ROUTE LEARNING**									
Immediate	error	1.29	1.14	5.21	2.08	<0.0001	−2.34	3.04	0.25
Delayed	error	0.07	0.27	0.64	0.74	0.01	−1.02	8.14	0.11

Note: fa, false alarm; SD, standard deviation; pd, proportional difference, $\frac{{\text{mean}}^{\text{old}}-{\text{mean}}^{\text{young}}}{{\text{mean}}^{\text{young}}}$;e^β^, multiplicative effect of age on error rate.

* Influential observation withheld

† effect of group, conditional on a covariate (see text)

‡ better performance in older adults.

### Mental navigation tests (TPPT)

#### Proximity judgments

Poisson regression on error count (collapsed across easy and difficult trials) revealed no significant effect of group, *p* = 0.88, even when taking into account frequency of visits, *p* = 0.86. It remains possible that older participants perform worse when number of years living in Toronto was taken into account, though this sample produced equivocal results, *X*^2^(1, *N* = 28) = 1.98, *p* = 0.16.

#### Distance judgments

ANOVA on mean deviation of estimates from actual distance (in km) revealed no significant effect of group, *p* = 0.2. Likewise, analysis of covariance (ANCOVA) revealed no significant effect of frequency of visits or number of years living in Toronto, *p* > 0.26 in both cases, even when an outlier was withheld from the analyses.

#### Vector mapping

ANOVA on deviation of estimates from actual distance (in km) revealed no significant effect of group, *p* = 0.15. ANCOVA revealed no significant effect of group when number of years living in Toronto was taken into account, *p* = 0.32, even when an outlier was withheld from the analyses. There was a marginally significant effect when frequency of visits was taken into account, *F*_(1, 26)_ = 3.92, *p* = 0.06, suggesting that the older adults performed better than the younger adults in estimating vector distance.

**Figure 1:**
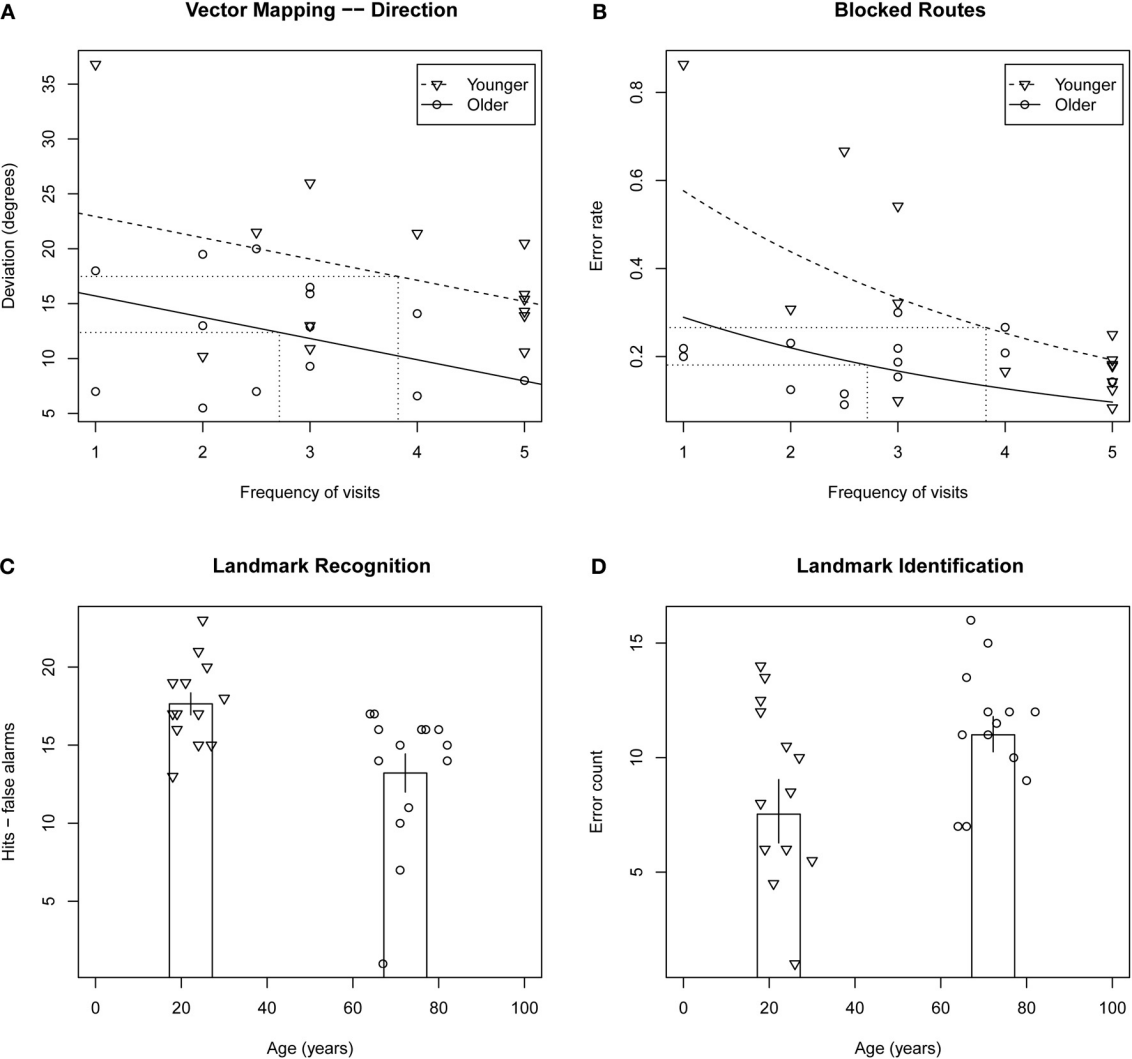
**Top row: scatterplots of mental navigation performance versus frequency of visits to Toronto for vector mapping—direction deviation (A) and blocked routes error rate (B), tasks on which older adults performed significantly better than younger adults.** Fitted models for younger and older adults are indicated by dashed and solid lines, respectively, and averages for the two groups are indicated by dotted lines. Bottom row: scatterplots of performance on landmark recognition **(C)** and landmark identification **(D)** versus age. Column height indicates average performance for each of the two groups. Error bars indicate ± one standard error.

ANOVA on mean deviation of estimates from actual direction (in degrees) revealed a significant effect of group, *F*_(1, 26)_ = 4.59, *p* = 0.04, such that older adults performed better than younger adults. ANCOVA revealed the effect of group to be larger when frequency of visits was taken into account, *F*_(1, 26)_ = 8.66, *p* = 0.007^3^. This result is presented in Figure 1A.

#### Landmark sequencing

Poisson regression on error count revealed no significant effect of group, *p* = 0.33, even when frequency of visits and number of years living in Toronto were taken into account.

#### Blocked routes

Poisson regression on error count when adjusting for the difference in total number of streets between participants revealed that the effect of group was marginal, *X*^2^(1, *N* = 28) = 3.18, *p* = 0.07, with older participants performing better than younger participants. This observed effect was enhanced when frequency of visits was taken into account, *X*^2^(1, *N* = 28) = 12.3, *p* < 0.0005 (see Figure 1B).

### Subjective re-experiencing

With respect to the participants' subjective report, significantly fewer older (7/14) than younger adults (14/14) reported adopting a first-person perspective during navigation in the blocked route task, Fisher's Exact Test, *p* = 0.006. More striking was the finding that 12/14 older adults but only 2/14 younger adults reported that their re-experiencing of the routes in memory lacked vividness and perceptual detail, Fisher's Exact Test, *p* = 0.0004.

### Landmark appearance

#### Landmark recognition

ANOVA on error count for hits minus false alarms revealed a significant effect of group, *F*_(1, 26)_ = 9.93, *p* < 0.004, with the older participants less able to discriminate between target and distractor landmarks than the younger participants (presented in Figure 1C). ANCOVA revealed the effect of group to remain even when frequency of visits and number of years living in Toronto were taken into account, *p* > 0.58 in both cases^4^. ANOVA performed for the sum of hits and false alarms was equivocal with respect to an effect of age group, *F*_(1, 26)_ = 2.06, *p* = 0.16, that was found to be significant when a participant with an unusually high number of false alarms was withheld, *F*_(1, 26)_ = 4.61, *p* < 0.04. The effect indicated a conservative response bias in the older adults and a non-conservative response bias in the younger adults.

#### Landmark identification

Poisson regression on error count revealed a marginally significant effect of group, *F*_(1, 25)_ = 3.32, *p* = 0.07, with the older participants identifying fewer Toronto landmarks than the younger participants^5^ (Figure 1D). There was no effect of frequency of visits or number of years living in Toronto.

### Baycrest route learning test

Poisson regression on error count for immediate learning of the new route (sum of runs 1 and 2) revealed an effect of group, *X*^2^ (1, *N* = 28) = 25.46, *p* < 0.0001, such that the older adults performed significantly worse than the young adults. The same was true for delayed learning of the route, *X*^2^ (1, *N* = 28) = 6.02, *p* < 0.01, with the older adults performing significantly worse than the young adults.

### Correlations

Correlational analyses indicated a positive correlation in new route learning between the two trials of the immediate condition, *r*_(26)_ = 0.56, *p* = 0.003, and a negative correlation between the rates of learning from the first to second trial of the immediate condition and from the immediate condition to the delay condition, *r*_(26)_ = −0.54, *p* = 0.004. New route learning in the delay condition was negatively correlated with landmark recognition for hits minus false alarms, *r*_(26)_ = −0.48, *p* = 0.01, and positively correlated for hits plus false alarms, *r*_(26)_ = 0.4, *p* = 0.04. Participants' subjective reports of navigation ability in new environments was positively correlated with their subjective reports of navigation ability in old environments, *r*_(26)_ = 0.55, *p* = 0.004, and negatively correlated with landmark recognition for hits minus false alarms, *r*_(26)_ = −0.42, *p* = 0.03. Correlations among the mental navigation tests of remote spatial memory were mostly positive, ranging from *r*_(26)_ = 0.43 to 0.57, *p* = 0.03 to 0.003. Finally, landmark recognition was positively correlated with landmark identification, *r*_(26)_ = 0.55, *p* = 0.004, whereas landmark identification was negatively correlated with vector mapping–distance, *r*_(26)_ = −0.55, *p* = 0.004, and landmark sequencing, *r*_(26)_ = −0.42, *p* = 0.03.

## Discussion

The current study investigated whether the changes in memory that accompany aging affect spatial representations formed long ago (i.e., 10 years ago or more). Older adults performed at least as well as younger adults on a wide range of mental navigation tests of remote spatial memory, even when amount of exposure to downtown Toronto was taken into account. No effect of age was found on proximity judgments, distance judgments, and landmark sequencing, and older adults outperformed younger adults on vector mapping and blocked routes. However, only half of the older adults, as compared to most of the younger adults, reported imagining the routes from a first-person perspective, and nearly all of the older adults, and hardly any of the young adults, reported that their imagined routes lacked perceptual richness and a feeling of re-experiencing. This age difference in self report was accompanied by significantly worse performance in the older adults in visually recognizing and identifying the landmarks for which intact spatial judgments had been made. Preserved performance in older adults was also in stark contrast to significantly worse navigation along a new route, both immediately after learning and following a 30-min delay. These results lend support to the view that the hippocampus is necessary for the establishment of spatial memories and for retaining and retrieving visual and experiential details even of representations formed long ago. However, it does not appear to be needed for representing the schematic attributes that are likely to have been extracted over many years navigating a large-scale environment.

Healthy aging is most often associated with a decline in memory and other cognitive processes, but some types of memory remain unchanged or even improve with age (e.g., Grady and Craik, 2000; Park and Reuter-Lorenz, 2009). In remote memory, episodic memory appears to be most vulnerable to the effects of aging, whereas semantic memory appears to resist such effects. Our work with humans and rats suggests similar distinctions in remote spatial memory. Here we showed that extensive navigation in a city environment over a long period of time leads to long-standing representations of spatial locations that resist disruption from aging. Comparable or better performance was found in old vs. young adults on a range of remote spatial memory tasks previously identified as more likely to be solved in an allocentric reference frame (proximity judgments, distance judgments, vector mapping) as well as those more likely to be solved in an egocentric reference frame (landmark sequencing, blocked routes; see Rosenbaum et al., 2004; Ciaramelli et al., 2010).

The current study does not provide direct evidence of a neuroanatomical substrate for remote spatial memory, but the results in the older adults closely resemble findings in patients with hippocampal damage or degeneration (Teng and Squire, 1999; Rosenbaum et al., 2000, 2005; Maguire et al., 2006). Patients with large bilateral medial temporal lobe (MTL) lesions that affect the hippocampus are able to negotiate their way in most places within premorbidly familiar environments and make a variety of judgments about the spatial relations contained within them. These findings suggest that, with sufficient time and experience, spatial memories can exist independently of the hippocampus and MTL. In the current study, correlations among the mental navigation tests did not appear to distinguish between tasks pre-classified as allocentric or egocentric, pointing to a blend of reference frames or a separate common strategy to sustain spatial memory performance in the face of other areas of cognitive decline associated with aging. These strategies, and resulting gist-like or schematic representations, may be supported by extra-hippocampal regions specialized for the initial coding of different information about environments within allocentric or egocentric frameworks, or in the integration or translation of the two frameworks. These regions include parahippocampal cortex within the MTL and regions of retrosplenial cortex and posterior parietal cortex to which the MTL regions are strongly interconnected (for a review, see Epstein, 2008). This possibility has been supported by evidence of co-activation of these regions during tests of spatial memory and navigation in neuroimaging experiments (Rosenbaum et al., 2004, 2007; Spiers and Maguire, 2006) and in studies with rats (Maviel et al., 2004; Frankland and Bontempi, 2005; Teixeira et al., 2006).

An alternative account of our findings is that both schematic and detailed, episodic-like aspects of remote spatial memories (discussed below) continue to depend on the hippocampus and that a gist is what survives following partial hippocampal damage. As mentioned, however, studies of patients with extensive damage to the hippocampus bilaterally indicate that even they can navigate in premorbidly learned environments and perform normally on tests of remote spatial memory similar to the ones included in the current study (Teng and Squire, 1999; Rosenbaum et al., 2000). Indeed, a functional neuroimaging study indicated that the little hippocampal tissue that remains in one such case (K.C.) was not differentially activated as he performed remote spatial memory tasks (Rosenbaum et al., 2007). Another possibility is that gist-like spatial memories rely on the hippocampus in older adults, as this structure is not severely damaged as is the case in the amnesic patients that have been studied. But, here again, neuroimaging studies indicate that even young adults do not differentially activate the hippocampus on these tasks (Rosenbaum et al., 2004).

Our results resemble findings in rats which showed that extensive experience in a complex maze as young rats enabled them to retain memory for efficient navigation to specific locations when they got old (Winocur et al., 2010a). Probe trials indicated that the rats' successful performance was, indeed, based on the application of allocentric spatial strategies and not on the use of non-spatial local cues or procedural learning. There are limits to how far we can extend this interpretation to older adults because there were some key differences. Unlike the older adults in the present study who performed normally on these spatial tasks, the aged rats performed slightly worse compared to when they were young. A possible account is that the older adults continued to visit downtown Toronto in recent years, whereas the aged rats were completely restricted from entering the environment for 15 months, about half a rat's lifespan. By contrast, older adults in the present study visited Toronto infrequently and significantly less often than did the young adults in the 5 years preceding the study. In fact, taking frequency of visits into account revealed better performance in the older than in the younger adults, suggesting that the older adults may have used a variety of non-hippocampal strategies to supplement their performance.

Findings of intact performance do not appear to be explained by the number of years that participants lived in Toronto, which was significantly greater for older than younger participants. There may be a minimum amount of experience and/or time (visiting once a week for no more than 10 years) needed for the formation and maintenance of a robust and presumably flexible representation of a real-world environment, but it appears that not much is gained beyond that minimum. Nevertheless, there are other potential confounds that we were unable to control or verify that relate to the nature of exposure to downtown Toronto. For example, the purpose of navigating in downtown Toronto (e.g., location of one's work, home, leisure activities), means of travel (walking, driving, taking public transit), and size or part of downtown Toronto in which one frequents may covary with age. These and other variables may influence the initial encoding and re-encoding of the environment as well as the quality of the representation itself.

Although age differences in representing spatial relations among landmarks and the routes between them were not apparent or favoured older adults, differences did emerge in subjective reports of the experiential quality of mentally navigating the routes. Whereas all of the young adults reported imagining the routes from a first-person perspective, half of the older adults reported a third-person perspective. Even more striking was the finding that the majority of the older participants reported that their re-experiencing of mentally navigating the route lacked vividness and richness of perceptual detail. Hirshhorn et al. (2011) reported a similar paucity of re-experiencing well-known Toronto routes in older adults who otherwise appeared to make accurate proximity judgments based on the same Toronto environment. Importantly, only re-experiencing of routes was correlated with autobiographical episodic memory and other neuropsychological tests of hippocampal function. Although we did not directly investigate the experiential and perceptual qualities of spatial judgments on vector mapping, a task that was also performed better by the older participants, it is possible that perceptual richness and re-experiencing interfered with efficient mental navigation in the younger participants.

The finding that aspects of mental navigation amid spatial locations were intact in the older adults contrasts with impaired recognition of landmarks that occupy those locations, reflecting both a difficulty discriminating between Toronto landmarks and similar-looking foil landmarks, and an overall conservative response rate. The data also suggested worse performance in older than younger adults in providing identifying information (name or other distinctive details) for landmarks that were accurately recognized as being located in Toronto. This additional finding suggests some modality-specific loss of distinctive visual information that enables naming. Object recognition impairment has also been reported in aged rats (Burke et al., 2010; see also McTighe et al., 2010), although these results may also reflect an age-related aversiveness to novelty. The recognition deficit appears to be independent of impaired spatial learning in the rats and resembles impaired pattern separation between stimuli that share visual features in rats and humans with perirhinal cortex lesions (Burke et al., 2011; for a review, see Graham et al., 2010). Similarly, the landmark recognition deficit described here resembles findings of impaired perceptual discrimination of complex scenes in relation to hippocampal and parahippocampal cortex lesions (Graham et al.). Indeed, we found a similar impairment of Toronto landmark recognition in a former taxi driver who had developed Alzheimer's disease (patient S.B.) in the context of reduced hippocampal and ventral visual cortex volumes (Rosenbaum et al., 2005). This contrasts with intact landmark recognition reported in the former taxi driver T.T. who was post-encephalitic and had bilateral hippocampal damage but intact perirhinal and parahippocampal cortices (Maguire et al., 2006). Park et al. (2004) and Schiavetto et al. (2002) have demonstrated that even when behavioural discrimination of faces, places, and other objects is not required, such object categories are not differentiated in ventral visual cortex to the same extent in old compared to young adults. It remains for future research to determine if the decline in landmark recognition is a consequence of such a lack of neural specificity.

It is possible that detailed knowledge of well-known city landmarks is not essential for navigation, especially when aerial views are presented to capture the landmarks in their entirety, and may, instead be treated as episodic-like details. It is unknown if visual recognition of landmarks is related to re-experiencing those landmarks and other visual features along an imagined route. In a recent study of remote spatial memory in patients with parietal lesions, we found a similar effect of age on the likelihood of reporting detailed, personal episodes associated with Toronto landmarks during a recognition task (Ciaramelli et al., 2010). Impaired autobiographical episodic memory for details of personal events was also found to co-occur with impaired landmark recognition in S.B. (Rosenbaum et al., 2005) and impaired recognition of neighbourhood houses in K.C. (Rosenbaum et al., 2000). In both patients, the impairment was in the context of intact remote memory for spatial locations but impaired learning of new routes. Findings that the perception of spatial elements and vivid recollections of those elements in remote memory are compromised in healthy and pathological aging and in amnesia suggest that the hippocampus is needed for linking different types of spatial details with each other and these to a rich episode.

Finally, we successfully replicated a consistent finding in the literature of impaired spatial learning in aging, which has been demonstrated in rats (Barnes, 1979; Gallagher and Pelleymounter, 1988; Winocur and Gagnon, 1998; Winocur et al., 2010a) and in humans, primarily based on virtual environments (Antonova et al., 2009; Head and Isom, 2010; Etchamendy et al., 2012; Rodgers et al., 2012; see Evans et al., 1984 and Cushman et al., 2008 for findings in real-world environments). The older adults in the current study committed significantly more errors than the young adults in the form of incorrect turns on two trials of an immediate learning condition as well as after a 30-min delay, though both groups showed improvement across trials. Interestingly, older participants seemed not to be cognizant of their deficient learning as they gave similar ratings of good navigational ability in both new and old environments. Both new route learning in the delay condition and ratings of navigational ability in new environments were negatively correlated with the ability to recognize well-known Toronto landmarks. Difficulties perceiving or encoding the appearance of newly encountered landmarks may have hindered route learning in the older adults, though work with rats described above would predict otherwise (see Burke et al., 2011).

Amnesia resulting from hippocampal damage is characterized by a profound inability to form and retain new spatial memories (e.g., Smith and Milner, 1981; Maguire et al., 1996, 2006; Holdstock et al., 2000; Rosenbaum et al., 2000; but see Corkin, 2002). This is supported by data from cellular recordings in animals (O'Keefe and Dostrovsky, 1971; Best et al., 2001; Moser et al., 2008) and humans (Ekstrom et al., 2003), animal lesion studies (Morris et al., 1982; Hampton et al., 2004; Lavenex et al., 2006), and human neuroimaging studies (Spiers and Maguire, 2006; Igloi et al., 2010). Findings like these contributed to the development of Cognitive Map Theory, which specifies that allocentric spatial memory for configural relations among objects located in the environment is uniquely dependent on the hippocampus (O'Keefe and Nadel, 1978; Bird and Burgess, 2008). This theory, and the related scene construction view of hippocampal function, accommodate findings in healthy aging, Alzheimer's disease, and hippocampal amnesia of impaired autobiographical episodic memory (Bright et al., 2006; Gilboa et al., 2006; Kirwan et al., 2008; Rosenbaum et al., 2008) and the construction of scenes and future events (Hassabis et al., 2007; Addis et al., 2011), which inherently involve a spatial framework (Byrne et al., 2007). The hippocampus is believed to sustain and guide this organizing principle, either by virtue of its purported role in processing spatial information or by some general binding process (Rosenbaum et al., 2009; Race et al., 2011). However, these theories do not take into consideration that detailed spatial memories change with time and experience and therefore cannot account for the preserved spatial memories that support navigation in older adults and other people whose hippocampal function is compromised.

Only the Transformation Hypothesis of spatial memory (Winocur et al., 2010b; Winocur and Moscovitch, 2011; see also Rosenbaum et al., 2001 and Moscovitch et al., 2005) can account for both the preserved and impaired spatial memories that we reported. The Transformation Hypothesis is based on the distinction between detailed or episodic-like spatial representations, which are impaired in aging and in hippocampal amnesia no matter how long ago the memories were acquired, and schematic/generic spatial representations of environments, which are resistant to the effects of aging and amnesia after they have been assimilated and stored over time. Spatial memories, like other types of declarative or relational memories, may change over time into a schematic or gist-like form. This process may resemble the “semanticization” of episodic memories in humans, whereby memory traces of repeated events become integrated with pre-existing knowledge in neocortex, stripped of contextual details that would allow for rich re-experiencing of the event. The hippocampus may be needed for the maintenance and retrieval of spatial details that are embedded within an episodic representation but not for a spatial layout that has been experienced in many different ways across a multitude of episodes. However, when fine discrimination is needed to distinguish routes or landmarks from one another, perirhinal cortex may also play a role even for remote memories of those routes or landmarks (Burke et al., 2011). Our findings provide evidence that older adults benefit from spatial-relational cues that are extracted over many different encounters with an environment as they do from the semantic gist that is extracted over repeated and varied experiences.

### Conflict of interest statement

The authors declare that the research was conducted in the absence of any commercial or financial relationships that could be construed as a potential conflict of interest.

## Footnotes

^1^The term “remote” is used to describe memories that are temporally distant and is not used in the current paper in reference to spatial distance.

^2^At the moment, we do not know how integrated the information on the map is and what kind of fine-grained information this kind of map can support. It is possible that this representation contains a series of local map-like representations or route knowledge that is strung together.

^3^Withholding data from an influential participant reduced the effect of group to marginal. This was true both without and with the frequency of visits covariate, *p* > 0.06 in both cases, with the effect continuing to be in the predicted direction.

^4^The age effect remained, even when withholding a potentially influential participant.

^5^Data were not available for one of the older adults.
